# The prevalent factors of anxiety in women undergoing mammography

**DOI:** 10.3389/fpsyt.2023.1085115

**Published:** 2023-09-18

**Authors:** Mohamed Ariff Jaafar Sidek, Kanchlla Amajid, Yi Sheng Loh, Muhammad Ariff Rosli, Iffah Syahirah Hashim, Nur Ashiqin Mohd Suffian, Norlia Abdullah, Marhani Midin

**Affiliations:** ^1^Department of Radiology, Faculty of Medicine, Universiti Kebangsaan Malaysia Medical Centre (UKMMC), Kuala Lumpur, Malaysia; ^2^Department of Surgery, Faculty of Medicine, Universiti Kebangsaan Malaysia Medical Centre (UKMMC), Kuala Lumpur, Malaysia; ^3^Department of Psychiatry, Faculty of Medicine, Universiti Kebangsaan Malaysia Medical Centre (UKMMC), Kuala Lumpur, Malaysia

**Keywords:** anxiety, mammography, prevalence, state-trait anxiety inventory, breast cancer

## Abstract

**Background:**

Breast cancer is the most common cancer among women in Malaysia. Anxiety is one factor that deters women from participating in mammography. This study aimed to assess the anxiety level and its associated factors in women undergoing mammography.

**Methods:**

A three-month cross-sectional study was conducted using self-administered questionnaires, encompassing socio-demographic details, clinical characteristics, and the State–Trait Anxiety Inventory.

**Results:**

The mean age of the participants was 57 years old (SD ±10.098). Repeat mammograms consisted of 48.8% of the participants. One-third (35.7%) of them had a history of breast disease. Most participants (84.5%) did not have a positive family history of breast cancer. The proportion of participants with moderate and high anxiety levels was 41.8%. The cause of anxiety was mainly due to the fear of the results (69%), while familiarity with the procedure reduced anxiety among respondents. Socio-demographic and clinical factors were not significantly associated with anxiety levels. However, a statistically significant positive correlation was found between state and trait anxiety scores (*r* = 0.568, *p* = 0.001, *n* = 213).

**Limitations:**

The urban setting and absence of questions on the location of origin in the study may have excluded data from the rural population. This may have prevented a true representation of the Malaysian population.

**Conclusion:**

The findings suggest a better understanding of the procedures involved as well as the subsequent disease management would be beneficial in alleviating anxiety prior to, during, and post-mammogram.

## 1. Introduction

Breast cancer is the most common type of cancer, being the highest cause of cancer-related death among women worldwide (1). The disease is also the most common form of cancer affecting Malaysian women. Approximately one out of 19 women in Malaysia are in danger of developing breast cancer in their lifetime, with the highest risk among those of Chinese ethnicity, followed by the local Indians and Malays (2).

The American Cancer Society Guidelines recommend that women from the age of 45 and above should subject themselves to annual mammograms as a preventive measure (3). When concerning early detection of breast cancer, the recommended screening methods are mammography, clinical breast examination, and breast self-examination, with the first being the most effective screening tool (4). Mammography is the only imaging technique that has a significant impact on the screening of asymptomatic individuals for breast cancer (5). A Swedish study has shown a 41% reduction in the risk of dying from breast cancer within 10 years, as a result of choosing to get early, preventive mammogram screening (6).

Despite the Malaysian Ministry of Health policy stipulating provisions for opportunistic mammography screening, uptake is varied and implementation remains a challenge (7). Most Malaysian women are reluctant to undergo mammography due to several reasons, including a fear of the positive result, harmful effects of radiation as well as pain related to the procedure (8). In a particular subsidized screening program in Malaysia, 11% of participants did not collect their reports while 10% were reluctant to undergo follow-up investigations (9). This highlights the lack of awareness and misconception of Malaysian women on the severity of breast cancer as well as the importance of early screening. Moreover, the lack of centralized cancer screening reporting has hampered efforts to effectively monitor and evaluate cancer control policies and programs, such as those related to mammograms and breast cancer (7).

The impact of anxiety on breast cancer patients extends from diagnosis, where it is one of the most common distressing consequences of mammography (10), right up to survivors of the disease, impacting their negative emotional state, ultimately affecting their quality of life (11). Furthermore, women who undergo mammography connect their mindset to several factors regarding breast cancer. Issues such as body image, social relationships, and sexual function lead to depression and anxiety, all interconnected which can become positive or negative coexistence in feelings and thoughts. Positive attitudes include adaptive and health-promoting behaviors while negative attributes include disgust with the appearance from the aftermath of surgery, low self-worth, emotional distress, and fear of cancer recurrence (12, 13). This emphasis on emotional well-being aligns with Durosini, Triberti’s (14) perspective on emotional abilities as pivotal factors for patients’ quality of life and health management. Understanding the effects and causes of anxiety could allow for tailored psychological coping mechanisms aimed at reducing its impact on the emotional response of patients, especially during mammography (15).

There has been little research done regarding the anxiety among women undergoing mammograms in Malaysia, despite the severe consequences of having a late check-up which may lead to a poorer prognosis. Hence, the present study aimed to determine the prevalence of anxiety in women undergoing mammography and the associated factors that may cause anxiety before, during, or after the procedure.

## 2. Methods

This study was a cross-sectional study conducted at the Radiology Department of Universiti Kebangsaan Malaysia Medical Centre (UKMMC), Cheras, Kuala Lumpur. The present study enrolled patients from the UKMMC radiology clinic, which operates Monday to Friday from 8:30 AM to 4:30 PM. Patients referred from breast clinics for breast cancer diagnosis follow-up and walk-in appointments from our screening program were included in the study.

### 2.1. Ethical issue

All respondents were required to read the informed consent form before filling out the questionnaire. The confidentiality of all participants was maintained throughout the study. The approval for this study was obtained from the Institutional Review Board, Research Ethics Committee, Medical Faculty, Universiti Kebangsaan Malaysia (reference code of UKM PPI/111/8/JEP-2016-356) before the commencement of the study. Permission to conduct the study at the Breast Imaging Centre in the Department of Radiology of UKMMC was also obtained from the Head of the Department of Radiology. License for the State–Trait Anxiety Inventory (STAI) had been purchased via the Mind Garden online portal, on behalf of Charles D. Spielberger, the developer and copyright owner of the STAI.^1^ The STAI had been translated into the Malay language and validated by Hashim, Hasyila (16). This version, along with the English version was utilized in the present study.

### 2.2. Sample size

The sample size was calculated using the formula for cross-sectional studies from Kish (17) with a prevalence (P) of 15, 95% confidence interval, and absolute precision of 5% (17). The estimated sample size for this study was 196 participants. Universal sampling was employed in this study. All mammography patients at the Radiology Department of UKMMC who met inclusion criteria were included in this study.

The inclusion criteria were women with mammography appointments, able to speak or understand the Malay or English language, and at least 18 years old. Participants were free to either use the Malay or English version of the questionnaire, whichever they were comfortable with. Reasons for exclusion were women who did not give consent for the study, omitted three or more items in the STAI questionnaire, or women who could not read or write.

### 2.3. Study instrument

The study instrument for this research was a set of self-administered questionnaires consisting of two parts. All the respondents were provided with a brief explanation of the study and written informed consent was subsequently obtained. Participants were then provided physical forms of the questionnaires to provide their responses. The process of filling in questionnaires was supervised by the authors to clear up any doubts they may have regarding the questionnaire. The filled questionnaires were then collected by the authors and stored securely under lock and key before data extraction was done. All data extraction and analysis were done only by the authors to ensure confidentially.

Part 1 consisted of socio-demographics, clinical characteristics of the patients as well as anxiety levels, while Part 2 was the Spielberger State–Trait Anxiety Inventory (STAI). Part 1(A) was on the socio-demographic characteristics of participants, consisting of age, race, educational level, marital status, and income level. Part 1(B) was on the clinical characteristics of patients which included: (i) any first-degree relatives who have been diagnosed with breast cancer; (ii) any previously diagnosed breast disease by a medical practitioner; (iii) diagnostic investigations; and (iv) knowledge regarding risk factors of breast cancer. Part 1(C) evaluated the anxiety level oneself, including: (i) the purpose of undergoing mammography; and (ii) reasons for anxiety when undergoing mammography.

The STAI in Part 2 of the questionnaire has been used extensively in research and clinical practice to diagnose anxiety and to distinguish from depressive syndromes (18, 19). It includes separate self-report scales for measuring state (S) and trait (T) anxieties. The S-Anxiety scale consisted of twenty statements that evaluated how respondents feel “right now, at this moment.” The T-Anxiety scale consists of twenty statements that assessed how participants felt in general. The STAI was designed to be self-administered, without any time limit for completion. Each STAI item was given a weighted score of 1 to 4. A rating of 4 indicated the presence of a high level of anxiety for ten S-Anxiety items and eleven T-Anxiety items. For the remaining ten S-Anxiety and nine T-Anxiety items, a high rating indicated the absence of anxiety. The weighted scores for the twenty items that made up each scale were added to obtain scores for the S-Anxiety and T-Anxiety scales. Anxiety scales varied from a minimum of 20 to a maximum of 80.

In this study, cut-off points of 20–39 were classified as low anxiety, 40–59 were classified as moderate anxiety, whereas 60–80 was classified as high anxiety. We further classified low anxiety as not anxious while moderate and high anxiety as anxious.

### 2.4. Statistical analysis

Obtained data were analyzed using Statistical Package for Social Science (SPSS) software version 22.0 (IBM Corp, NY, United States). The numerical data such as age, state, and trait anxiety score were described with mean and standard deviation (SD). Frequency and percentage were used to describe categorical data such as demographic factors, clinical characteristics, reasons for anxiety as well as anxiety levels among participants. Association between independent variables and anxiety level were tested using Pearson’s Chi-Square test. An Independent *T*-test was employed in comparing means between two independent variables where one was quantitative (age) and the other a dichotomous qualitative variable (anxiety level). The association between numerical independent variables (state anxiety score) and numerical dependent variables (trait anxiety score) was determined using the Pearson correlation. The level of significance was set at a 95% confidence interval with a *p*-value < 0.05.

## 3. Results

A total of 221 respondents were selected to participate in the present study. However, only 213 respondent’s data were included in the analysis due to incomplete feedback. Table 1 gives a summary of the socio-demographic characteristics of respondents. The age of respondents ranged from a minimum of 31 to a maximum of 81 years of age. Most of the respondents were of Malay ethnicity (53.1%). About half of them received an education up to secondary school (51.2%) with more than three-quarters of the respondents being married (79.8%). A majority of the respondents had a low monthly income (67.6%).

**Table 1:** Socio-demographic and clinical characteristics of the participants.

Variables	Frequency (%) (*n* = 213)
**I. Socio-demographic characteristics**	
**Age (years)**	
Mean = 57.26 (SD = 10.098)	
**Race**	
Malay	113 (53.1)
Chinese	69 (32.4)
Others	31 (14.6)
**Education**	
No formal education	6 (2.8)
Primary education	38 (17.8)
Secondary education	109 (51.2)
Tertiary education	60 (28.2)
**Marital status**	
Single	43 (20.2)
Married	170 (79.8)
**Income level**	
Low (<RM3000)	144 (67.6)
Middle (RM3000-10000)	64 (30.1)
High (>RM10000)	5 (2.3)
**II. Clinical characteristics**	
**History of breast disease**	
Yes	76 (35.7)
No	137 (64.3)
**Family history of breast cancer**	
Yes	33 (15.5)
No	180 (84.5)
**Purpose of mammography**	
Screening	89 (41.8)
Diagnose	20 (9.4)
Follow-up	104 (48.8)
**Frequency of undergoing mammography**	
First time	40 (18.8)
Multiple times	173 (81.2)
**Result of the mammography**	
Benign breast diseases	166 (77.9)
Breast malignancy	47 (22.1)

Income levels were determined based on the accepted Malaysian urban poverty line, rounded up (20, 21).

More than half of the participants (64.3%) did not have any history of breast disease before mammography, while 35.7% had previously been diagnosed with benign breast diseases. The majority (84.5%) did not have a positive first-degree family history of breast cancer. As for the purpose of screening, 48.8% came for follow-up treatment, followed by 41.8% who came for screening purposes while 9.4% underwent mammography to get a definite diagnosis of their conditions.

During the data collection period, we found out that 81.2% of the participants had already undergone mammography multiple times while the remaining 18.8% were first-timers. Based on the results of the mammography, the majority of them (77.9%) were diagnosed to have benign breast diseases and 22.1% were found to have breast malignancies.

**Figure 1:**
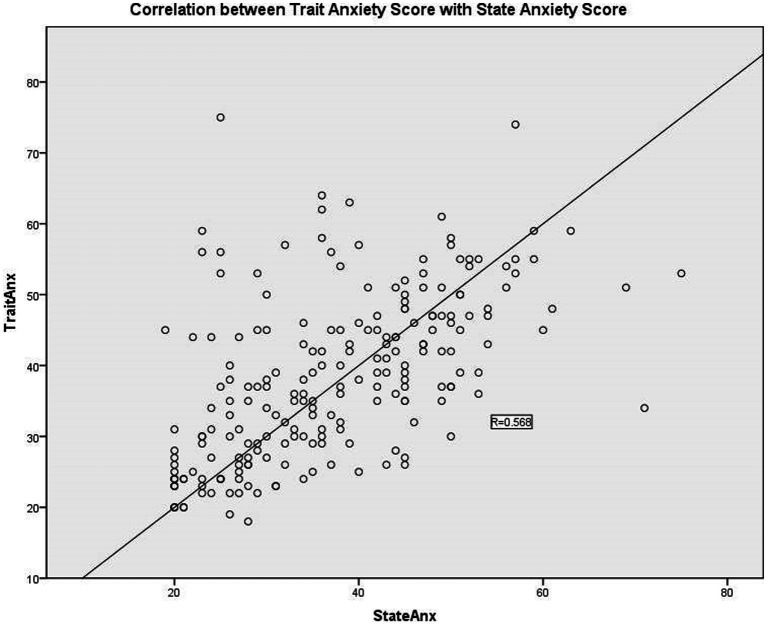
Correlation between state anxiety score with trait anxiety score.

The mean values of state and trait anxiety levels among the participants were 37.15 and 38.4, respectively (Table 2). Low anxiety level was recorded in 58.2% of respondents, while the remaining 41.8% were of moderate to high anxiety level. There was a moderately strong, positive correlation between state and trait anxiety scores, which was statistically significant (*R* = 0.568, *N* = 213, *p* < 0.001), as shown in Figure 1.

**Table 2:** Mean anxiety score of respondents.

Variables	Mean ± SD
**Anxiety score**	
State-anxiety	37.15 ± 11.078
Trait-anxiety	38.4 ± 11.726
**State anxiety level**	Frequency (%)
Low anxiety	124 (58.2)
Moderate-high anxiety	89 (41.8)

**Figure 2:**
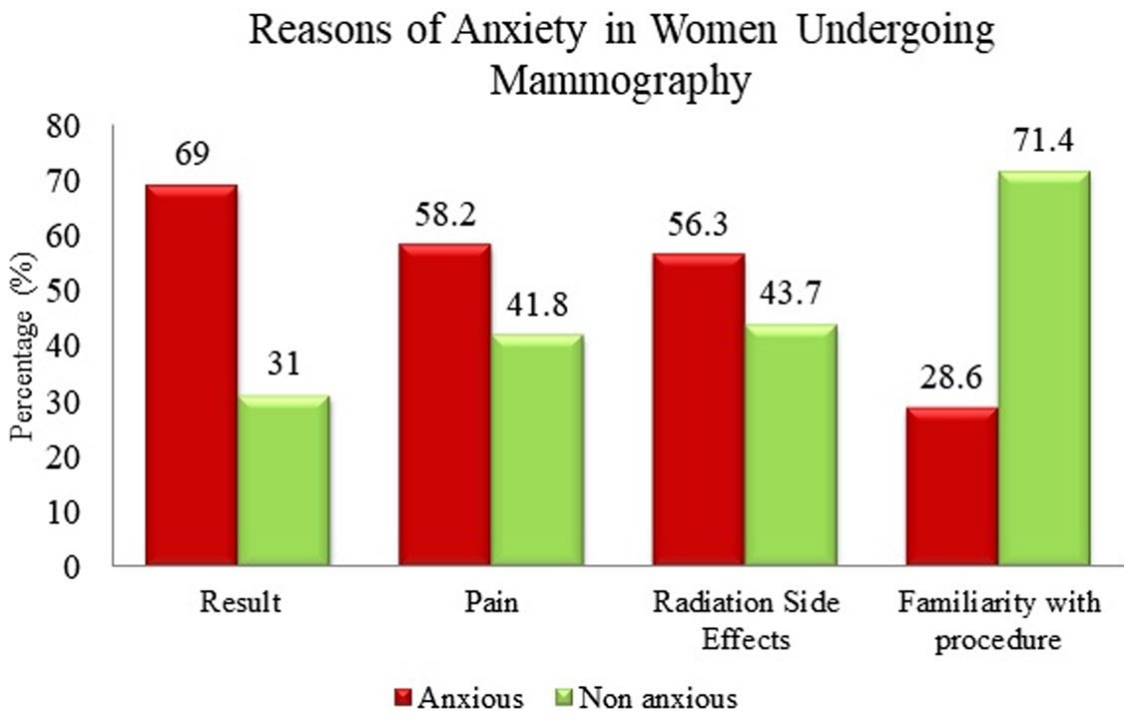
Reasons for anxiety in women undergoing mammography.

The reasons for anxiety among the respondents included the results, pain during the procedure, side effects of the radiation, and familiarity with the procedure. Participants were allowed to pick more than one option for the reason of anxiety (Figure 2).

More than half of the respondents (69%) had increased anxiety due to awaiting the results of mammography. Among all the reasons for anxiety, fear of results due to mammography has the highest number of respondents, 147 out of 213. Out of 213 respondents, 124 had anxiety due to pain during the procedure (58.2%). One hundred twenty respondents had anxiety due to the side effects of the radiation during the mammography, which constituted about 56.3% of the total respondents. Familiarity with the procedure of mammography caused the least anxiety in the respondents (28.6%).

The association between socio-demographic factors and anxiety was not statistically significant. Referring to Table 3, all tested factors were not significantly associated with anxiety (*p*-value > 0.05). The age of anxious respondents was not associated with levels of anxiety (*p*-value = 0.580).

**Table 3:** Association between all variables and anxiety level.

**I. Association between socio-demographic factors with anxiety level**				
Variables	Anxiety		X^2^	*p*-value
	Anxious *n* (%)	Not anxious *n* (%)		
**Age** (mean age ± SD)	56.81 ± 10.16	57.59 ± 10.09		0.580
**Race**				
Malay	47 (52.8)	66 (53.2)	0.204	0.903
Chinese	30 (33.7)	39 (31.5)		
Others	12 (13.5)	19 (15.3)		
**Educational level**				
No education and primary	21 (23.6)	23 (18.5)	0.944	0.624
Secondary	45 (50.6)	64 (51.6)		
Tertiary	23 (25.8)	37 (29.8)		
**Income level**				
<RM3000	57(64)	87(70.2)	0.885	0.347
≥RM3000	32(36)	37(29.8)		
**Marital status**				
Single	21 (23.6)	22 (17.7)	1.102	0.294
Married	68 (76.4)	102 (82.3)		

**II. Association between clinical characteristics with anxiety level**				
Variables	Anxiety levels		X^2^	*p*-value
	Yes *n* (%)	No *n* (%)		
**Family history of breast cancer**				
Yes	15 (7.0)	18 (8.5)	0.216	0.642
No	74 (34.7)	106 (49.8)		
**History of benign breast disease**				
Yes	28 (13.1)	48 (22.5)	1.186	0.276
No	61 (28.6)	76 (35.7)		
**Purpose of mammography**				
Screening	38 (17.8)	51 (23.9)	3.543	0.170
Diagnosis	12 (5.6)	8 (3.8)		
Follow-up	39 (18.3)	65 (30.5)		

There was also no statistically significant association between clinical factors and state anxiety levels among women undergoing mammography (*p* > 0.05), as shown in Table 3.

## 4. Discussion

From the data analysis, the mean age of the respondents was 57.26 (SD ± 10.098). This was consistent with Yip et al., where the incidence rate of breast cancer was highest in the 50–59 year age group, especially among the local Malay and Chinese women (22). In terms of ethnic distribution, most of the respondents in the present study were of Malay ethnicity, followed by Chinese with the least being from other races. A report by the Department of Statistics of Malaysia on the total population by ethnic group in Ulu Langat reflected similarities with our data in terms of ethnic distribution. Ulu Langat is the neighboring district from where the study centre is located. The Malays and other indigenous groups, (57.29%) were the major ethnic group staying in Ulu Langat, whereas the Chinese (31.45%) and other races (11.26%) belonged to the minor ethnic groups (23).

A majority of the respondents attained secondary education, while one-third of them managed to complete tertiary education. This was parallel to the data from the Department of Statistics on Education and Social Characteristics in 2010, in which 21.6% of Malaysian citizens aged 20 years and older managed to attain tertiary education (24). In our study, the majority of respondents were married. In a survey conducted by the Ministry of Health in 1996, there was a significantly higher screening rate in married women as compared to other marital categories of women (23).

The majority of our respondents were from the low-income brackets, while about one-third of them had middle income, with only a minority having a high income. The higher prevalence of lower and middle-income demographics may be due to the study centre being a government healthcare facility, which provides subsidized healthcare. In most cases, only a nominal fee is collected from patients at Malaysian healthcare facilities. Moreover, government healthcare schemes, such as Iltizam Selangor Sihat or Selangor Saring, provide free screening for non-communicable diseases in general, which includes mammogram screenings for breast cancer (25, 26). As such, affordability should not be an issue with getting screening; awareness of such schemes may be a limiting factor instead.

More than half of the participants did not have any history of breast disease before the mammography, with a majority not having a positive first-degree family history of breast cancer. Despite being negative for these two factors, their presence in mammography screening could be attributed to an increase in awareness of the importance of early detection. The non-first-timers made up 81.2% of the participants for mammography. In our opinion, these repeat patients could have been much more familiar with the procedures involved in mammography due to their experience, thus leading to lower levels of anxiety; which was also seen in the present study, where familiarity caused the least anxiety.

The mean for the state of anxiety scores among women undergoing mammography was 37.15, which was categorized as low anxiety. This shows that most of the women were not anxious before undergoing mammography screening. A statistically significant, positive correlation was present between the state and trait anxiety scores. This association was expected as the STAI manual did state that high-state anxiety is expected in the presence of high-trait anxiety (27).

The largest group of respondents had increased anxiety while waiting for the results (69%); most of them had a fear of getting a positive result. Secondly, anxiety due to pain during mammography was also high which accounted for 58% of the respondents. Relevant health education on the importance of early detection concerning breast cancer is imperative in reassuring women of the minimal downsides of the procedure. Accurate information on the small radiation risk and pain during the procedure may help to alleviate fears about mammograms. This is consistent with the findings of past studies which have indicated that women undergoing mammography experienced less pain/discomfort and anxiety when enlightened with pertinent information through a knowledgeable nurse (28, 29).

In this study, we found that there was no significant association between age, race, marital status, income level, or educational level with anxiety. Although studies such as Yu et al. managed to highlight a link between age and anxiety (30), others such as Chojniak et al. were consistent with our study (31). The lack of significance between marital status and anxiety seen in the present study was consistent with, Chojniak et al. (31) and Bolukbas et al. (32).

Educational levels were also not significantly associated with anxiety, which contradicted a few studies in which women with low educational levels had higher levels of anxiety (30, 32, 33). The contrasting findings could be attributed to the awareness campaigns done by various government and non-governmental organizations (NGOs), including the Ministry of Health and National Cancer Society Malaysia, making them less anxious during the procedure (34–36). Additionally, as the majority of our participants had previously undergone the procedure at least once, familiarity with the procedure itself may have contributed to the lack of correlation between education level and anxiety levels.

There was no significant association between having a family history of breast cancer and anxiety level, similar to a study done by Chojniak et al. (31). Our findings were contrary to those seen by Guvenc et al. and Greco et al. who stated that women with a family history of breast cancer had more anxiety to undergo mammography than the opposite (37, 38). Those who have relatives with breast cancer may have the wrong perception that they may be susceptible to the disease, leading to an increased level of anxiety (37). This discordance between these studies and ours may be because most of our participants had undergone mammography multiple times. Furthermore, women who have a history of breast cancer in the family would have more information on breast cancer and awareness of screening tests than other women (38, 39).

In addition to the above, we also found that having a history of breast disease was not significantly associated with the level of anxiety in women who undergo mammography. This insignificance may be due to most of our participants having benign results in the past that helped to reduce their anxiety levels. This is in accordance with the findings by Guvenc et al. (38), whereby those who had a history of breast cancer had higher anxiety levels when undergoing mammography compared to those who had benign breast disease diagnoses (38).

The purpose of doing mammography for screening, follow-up, or diagnosis, was not significantly associated with the state of anxiety. This was consistent with other studies that also could not prove any association between the purpose of mammography and the state of anxiety level in these women (31, 32).

The prevalence of anxiety in breast cancer patients in UKMMC was 31.7%, as previously reported by Hassan et al. (40). However, we could not find an association between breast conditions with anxiety in the present study. This finding was also consistent with studies by Chojniak et al. (31), Bolukbas et al. (32), and Brunton et al. (33). Being diagnosed with breast cancer did not cause a higher level of anxiety as some patients might respond differently after knowing the results, especially among those who had good self-perception about the breast cancer cure rate.

Bolukbas et al. stated that women who had multiple mammography visits had a significantly higher level of anxiety (32). Women with multiple mammography visits were anxious because they thought that the doctor was suspecting cancer and asked them to do regular screening. However, we could not find any association between these two variables. This is probably because women who underwent multiple mammographies previously may have good knowledge and experiences about the procedure, leading to them being less anxious.

Awareness and willingness of women to undergo screening and regular mammogram examinations will be able to reduce the morbidity and mortality of breast cancer (6).

## 5. Limitations

There were a few limitations that we encountered that could have affected the outcomes. Although we did manage to exceed the intended sample size, the data obtained may not have been a true representation of the population. A prevalence of 15% was used during the sample size calculation, differing from the norm where a prevalence of 40 to 50% was used instead. This is due to our limited funding and time frame for this study to be completed within 3 months. Moreover, as the study centre was focused in an urban setting and localized to one specific locality, the findings could not be extrapolated to a wider population. The difference in demographics and exposure to knowledge on breast cancer will differ between those in an urban from a rural setting. This may influence the levels of anxiety. One of the major barriers highlighted in preventing widespread mammogram screening in Malaysia has been attributed to a lack of awareness and access to equipment in rural areas (7). Rural populations make up 25% of the local population (41).

Secondly, the original STAI did not provide a specific cut-off point for the different levels of anxiety. As such, there may have been discrepancies between our findings and previous studies, be it among the local or international populations. This would make effective comparisons quite difficult.

In the present study, the STAI questionnaire was used as the primary tool to assess anxiety levels in women undergoing mammograms. While the STAI questionnaire possesses established reliability and validity in a clinical setting as mentioned by Gustafson et al., we acknowledge the potential limitations of using a single assessment method in capturing the complexity of anxiety experienced. Given practical constraints, the present study was unable to incorporate additional measures. There is potential value in future research exploring a multi-method approach to provide a more comprehensive understanding of anxiety during mammographic procedures.

## 6. Conclusion

This study managed to show that there was no significant relationship between sociodemographics and clinical variables. Interestingly, we managed to highlight the highest cause of anxiety was due to anticipation of a possible malignant finding while familiarity with the procedure had the opposite effect. A comprehensive awareness of what to expect before, during, and after the procedure seems to be the best way to alleviate anxiety among those undergoing mammogram screening.

We also managed to show a moderately strong, positive correlation between state and trait anxiety scores, which was statistically significant. This implies that a person with high trait anxiety is more likely to have higher state anxiety compared to another person with low trait anxiety.

Further research should involve patients from different hospitals in Malaysia to provide additional insight into the association between anxiety and its contributing factors in women undergoing mammography. Understanding the associated factors that contribute to increased anxiety may provide insights that could lead toward tailored counter-measures; hopefully increasing the number of Malaysian women undergoing mammography.

## Data availability statement

The raw data supporting the conclusions of this article will be made available by the authors, without undue reservation.

## Ethics statement

The studies involving humans were approved by the Institutional Review Board, Research Ethics Committee, Medical Faculty, Universiti Kebangsaan Malaysia. The studies were conducted in accordance with the local legislation and institutional requirements. The participants provided their written informed consent to participate in this study. Written informed consent was obtained from the individual(s) for the publication of any potentially identifiable images or data included in this article.

## Author contributions

MAJS: validation, review, and editing. KA, ISH, and NAMS: investigation and data curation. MR: software and formal analysis. YSL: methodology and original draft preparation. NA and MM: conceptualization, review, and editing. All authors contributed to the article and approved the submitted version.

## Funding

This research was funded by the Universiti Kebangsaan Malaysia Medical Centre (UKMMC) (Grant No. FF-2016-253).

## Conflict of interest

The authors declare that the research was conducted in the absence of any commercial or financial relationships that could be construed as a potential conflict of interest.

## Publisher’s note

All claims expressed in this article are solely those of the authors and do not necessarily represent those of their affiliated organizations, or those of the publisher, the editors and the reviewers. Any product that may be evaluated in this article, or claim that may be made by its manufacturer, is not guaranteed or endorsed by the publisher.

## Footnotes

^1^ https://www.mindgarden.com/
